# Effects of drinking water valerian extract and stocking density on stress, performance, and egg quality in laying Japanese quails

**DOI:** 10.1016/j.psj.2026.106884

**Published:** 2026-03-30

**Authors:** Sidney Huapaya, Daniel Huaringa, Rubén Cespedes, Liliana Jiménez, Otto Zea, Iván Meza, Carlos Vílchez-Perales

**Affiliations:** a Departamento de Nutrición, Facultad de Zootecnia, Universidad Nacional Agraria La Molina, Lima 15024, Perú

**Keywords:** Valeriana officinalis, Cage density, Laying performance, Stress biomarkers, Japanese quail

## Abstract

The objective of this study was to evaluate the effects of Valerian (*Valeriana officinalis L.*) extract added to drinking water on productive performance, egg quality, and stress indicators in laying quails (*Coturnix japonica*) under different stocking densities, with emphasis on their interaction with valerian supplementation. A total of 180 females, 9 weeks of age, were randomly allocated to cages and assigned to two Valerian extract levels (0 and 4 mg/mL of water) and three stocking densities (1, 2, or 3 birds per cage) for seven weeks. Productive performance (body weight, feed intake, laying rate, and egg mass), external and internal egg quality traits, tonic immobility duration, heterophil-to-lymphocyte ratio, corticosterone concentration, and haematological parameters were evaluated. Valerian extract supplementation did not affect productive performance or overall egg quality (*p* > 0.05). Feed intake was influenced by stocking density (*p* < 0.05), being lowest in singly housed birds, while shell thickness was reduced in quails receiving Valerian extract. Valerian supplementation significantly reduced corticosterone concentration (from 0.62 to 0.38 µg/dL; *p* < 0.01) and numerically lowered the heterophil-to-lymphocyte ratio (from 1.52 to 1.15; *p* = 0.078), whereas higher stocking densities tended to increase stress indicators. Significant Valerian × density interactions were observed for shell thickness, albumen height, yolk height, yolk color, heterophil-to-lymphocyte ratio, haematocrit and eosinophil counts, and stocking density affected monocyte percentages (*p* < 0.05). In conclusion, Valerian extract administered via drinking water reduced physiological stress indicators without affecting productive performance or egg quality under the evaluated stocking densities.

## Introduction

Livestock production in general and domestic poultry production in particular plays a vital socio-economic role for people living in low-income countries (Mohamadinejad et al., 2024; Khezri et al., 2025; Mohammadabadi et al., 2025a). Domestic poultry are widely distributed avian species around the world, due to their short generation interval and adaptability in a wide range of agro ecologies (Mohammadifar and Mohammadabadi, 2017; Khabiri et al., 2025; Mohammadabadi et al., 2025b). The domestic poultry provide high quality protein and income for the poor rural households and are the most widely kept livestock species in the world (Mohammadabadi et al., 2010; Mohammadifar and Mohammadabadi, 2018; Mohammadabadi et al., 2024). This is due to the presence of the valuable traits of poultry like disease resistance, adaptation to harsh environments and ability to utilize poor quality feeds (Shahdadnejad et al., 2016; Khabiri et al., 2023; Mohammadabadi et al., 2025c).

Stress in laying birds has a direct impact on egg production, causing significant economic losses for poultry producers. Factors such as high temperatures, high stocking densities, and inadequate management practices are major causes of stress in these animals. Birds are characterized by having very limited body resources for growth, reproduction, responses to environmental changes, and defense mechanisms compared with mammals (Rosales, 1994). Consequently, any slight deviation from normal conditions leads to a rapid redistribution of body resources, including energy and proteins, at the expense of growth, reproduction, and health (Beck, 1991; Brake, 1985; Gross and Siegel, 1981). When these challenges become more intense or frequent at a given time, severe chemical and physical changes occur within the birds, resulting in weakness and fatigue. Such conditions can lead to starvation and infectious diseases (Freeman, 1987; Dohms and Metz, 1991).

It is well known that when stress factors are present, they lead to an increase in the release of glucocorticoids, such as cortisol or corticosterone. These hormones, in turn, alter the function of numerous tissues to mobilise, store, or conserve energy to meet the demands imposed by stress. Among the various processes affected by cortisol are glucose metabolism, bone metabolism, and cardiovascular and immune functions. The central nervous system is responsible for controlling cortisol levels in the blood, which are elevated in response to stress and to low concentrations of circulating glucocorticoids (Gómez and Escobar, 2006). Previous studies have reported that the inclusion of herbal or plant-based additives can improve laying performance, physiological responses and egg quality in quails (Gravena et al., 2009; Marqués, 2010; Utami and Akbar, 2025; Wuthijaree et al., 2025).

Valerian (*Valeriana officinalis L.*) is a well-known medicinal plant traditionally used since ancient times. Its root exhibits sedative and hypnotic activity and is therefore recommended in cases of nervousness or anxiety and in sleep disorders. It has also been demonstrated to possess spasmolytic and muscle-relaxant effects. Phytochemical analyses have shown that this plant contains high concentrations of valepotriates, of which valtrate is perhaps the most biologically active compound within this group (Castillo et al., 2002). It has been suggested that these effects might also be attributable to valerianic acid or to the baldrinals themselves (Consejo general de colegios oficiales de farmacéuticos, 2013). The mechanism of action of valerian is complex and not yet fully elucidated, although it may act at both pre- and postsynaptic levels. In vitro assays have demonstrated that valepotriates inhibit GABA transaminase, thereby reducing the degradation of γ-aminobutyric acid (**GABA**). Furthermore, they may increase its release into synaptic spaces and decrease its reuptake. In addition, large amounts of glutamine have been detected in valerian, which may be taken up by neurons and converted into GABA.

Few studies have evaluated the use of valerian (*Valeriana officinalis* L.) in poultry species, particularly with respect to its effects on stress-related responses. For instance, Gravena et al. (2009) reported that dietary inclusion of valerian in Japanese quails did not significantly affect productive performance, although potential behavioural and physiological effects were suggested. In broiler chickens, valerian extract (**VE**) administered through drinking water has been shown to influence neurobiomarkers associated with stress, including increased activity of GABAergic and serotonergic systems, supporting its sedative and modulatory role (Maty et al., 2025). In addition, supplementation with a herbal mixture containing valerian (10 % inclusion within the extract) has been reported to reduce stress indicators such as corticosterone levels and heterophil-to-lymphocyte ratio in broilers (Skomorucha and Sosnówka-Czajka, 2018). Similarly, valerian in combination with *Moringa oleifera* has been evaluated as a natural additive in broiler chickens, reporting effects on productive parameters (Palacios and Solis, 2018). These findings indicate that valerian and valerian-containing phytogenic formulations may exert their effects through modulation of neuroendocrine pathways associated with stress. However, the available evidence remains limited and inconsistent, particularly regarding its effects on productive performance and egg quality, and there is scarce information on the combined effects of valerian extract and stocking density in laying quails.

Despite extensive research on stress in laying birds, limited information exists on the effects of valerian supplementation on stress indicators, productive performance, and egg quality in quails. The present study was conducted to evaluate the effects of adding VE to the drinking water, together with different stocking densities, on productive performance, egg quality, stress indicators, and blood biochemical parameters in laying quails, providing new insights into the use of a natural plant extract to mitigate stress and improve production outcomes.

## Materials and methods

The study complied with the Guide for the Care and Use of Agricultural Animals in Research and Teaching, 4th edition, Chapter 9: Poultry (ASAS, 2020).

### Animals and experimental design

The experimental trial was conducted at the Poultry Research Unit of the Universidad Nacional Agraria La Molina, Lima-Peru. A total of 180 female Japanese quails (*Coturnix japonica*) at the early laying stage (9 weeks of age) were distributed into four experimental batteries. Each battery consisted of five tiers, with five individual cages per tier. The lower tier of two batteries was not used, resulting in a total of 30 cages per treatment

A commercial Valerian Extract (VE) derived from the medicinal plant *Valeriana officinalis L.* was used and administered through drinking water (0 and 4 mg/mL of water; Factor A). The product consisted of a lyophilized root extract of *Valeriana officinalis L*., formulated in vegetable glycerin with alpha- and gamma-cyclodextrins. According to the declared composition, the product provided, per millilitre, 180 mg of valerian extract, 60 mg of gamma-cyclodextrins, 30 mg of alpha-cyclodextrins, and 0.28 mg of valerenic acid. Birds were also subjected to three stocking densities (1, 2, and 3 quails per individual cage; Factor B). The dimensions of the individual cages were 23 × 15.5 × 21 cm. Each cage was equipped with trough feeders and cup drinkers.

The experiment was conducted using a completely randomised design with a 2 × 3 factorial arrangement, resulting in six experimental treatments: T1 (0 mg VE, 1 bird/cage), T2 (0 mg VE, 2 birds/cage), T3 (0 mg VE, 3 birds/cage), T4 (4 mg VE, 1 bird/cage), T5 (4 mg VE, 2 birds/cage), and T6 (4 mg VE, 3 birds/cage). Each treatment was replicated three times, and the cage was considered the experimental unit. The sample size (180 quails) was selected based on previous experimental studies in laying quails that used the same number of birds to evaluate productive performance and physiological responses (Gravena et al., 2009; Huapaya et al., 2019; Saleh et al., 2020; Mustafa et al., 2024; Hariono et al., 2025).

Both the control and VE-treated groups were managed under identical water-restriction conditions. Drinking water was offered in a fixed daily volume of 75 mL per bird, fractionated into two equal allotments per day, for all experimental groups. The control group received drinking water without VE, whereas the experimental group received water supplemented with VE. For VE administration, 6.75 L of drinking water were prepared daily for the experimental group (90 quails) by diluting five commercial bottles (30 mL each) of the VE, resulting in a final concentration of 4 mg VE/mL. Water was supplied in controlled volumes to ensure complete consumption. Based on the standardized daily water allowance, the estimated VE intake was 300 mg per bird per day, consistent with previous poultry studies using oral administration of commercial valerian extract to evaluate production and stress-related parameters (Ramírez et al., 2015; Huapaya et al., 2019). Drinking water for all experimental groups was chlorinated daily at a dose of 0.1 mL of 4 % sodium hypochlorite per litre and allowed to stand for 24 h prior to use to avoid potential interference with the composition of the VE. Water was renewed daily at 08:00 h for both the experimental and control groups. The quails were fed a basal corn–soya diet (Table 1), formulated to meet or exceed the nutritional requirements of laying quails according to the NRC (1994) and its nutritional composition was calculated based on ingredient composition tables and formulation software. The basal diet contained standard non-experimental feed additives commonly used in commercial poultry formulations to ensure nutritional adequacy, maintain feed stability, and support gastrointestinal health; these components were uniformly applied across all treatments. A daily allowance of 25 g of feed per bird was offered, and leftover feed was weighed weekly to record actual daily feed intake. Feed allowance was controlled to standardize nutrient intake and avoid excessive feed wastage associated with *ad libitum* feeding in caged quails. The trial lasted seven weeks (including one week of adaptation, during which VE was not added to the drinking water). The average ambient temperature during the experimental period was 30°C, with a maximum of 34°C and a minimum of 26°C. Mortality and unproductive recorded during the adaptation period were replaced before the start of data collection

**Table 1:** Composition and nutritional value of the diet.

Ingredients	Inclusion (%)
Corn	57.07
Soybean meal	23.80
Wheat bran	1.30
Fish meal	6.00
Soybean oil	2.12
DL-Methionine	0.18
Calcium carbonate	7.28
Monocalcium phosphate	1.34
Salt	0.15
Quail vitamin–mineral premix*	0.10
Choline chloride	0.07
Mycotoxin binder	0.20
Antifungal agent	0.10
Bicarbonate	0.15
Antioxidant	0.02
Sodium butyrate 70%	0.10
Enzyme blend**	0.02
Calculated nutrient composition	
ME (Kcal/kg)	2980
Crude protein (%)	20.00
Lysine (%)	1.00
Methionine (%)	0.48
Met + Cys (%)	0.72
Threonine (%)	0.74
Calcium (%)	3.48
Available phosphorus (%)	0.40
Sodium (%)	0.15

⁎ Per kg of premix, provided vitamin A 3.30 mIU, vitamin D3 0.90 mIU, vitamin E 25 kIU, vitamin K3 1.0 g, thiamin 2.0 g, riboflavin 4.0 g, pyridoxine 3.0 g, niacin 20.00 g, folic acid 1.0 g, cyanocobalamin 3 mg, pantothenic acid 15 mg, biotin 15 mg, Zn 50 g, Cu 5 g, I 0.30 g, Fe 60 g, Mn 60 g, and organic Se 0.20 g.

⁎⁎ The diet was supplemented with phytase (0.01 %) and xylanase (0.01 %). Enzymes were added on top and were not considered in the nutrient matrix of the diet.

### Measured variables

The variables analyzed included productive performance parameters, such as body weight (**BW**), feed intake (**FI**), and laying rate (**LR**). The variables evaluated to determine egg quality were grouped as follows; external characteristics: egg weight (**EW**), egg length **(EL**), egg diameter (**ED**), shell weight **(SW**), and shell thickness (**ST**) and internal characteristics: albumen diameter (**AD**), albumen height (**AH**), albumen weight (**AW**), yolk diameter (**YD**), yolk weight (**YW**), yolk height (**YH**), yolk color (**YC**), Haugh Unit (**HU**), and yolk index (**YI**). Egg mass (g/bird/d) was calculated as the product of EW and LR. For these variables, data were averaged per cage to account for the shared housing of birds.

Stress indicators included serum corticosterone, heterophil-to-lymphocyte (**H:L**) ratio, and tonic immobility time. In addition, haematological parameters were evaluated, including haematocrit, haemoglobin, red blood cells (erythrocytes), white blood cells (leucocytes), heterophils, lymphocytes, monocytes, and eosinophils. For blood parameters, each bird was considered an independent experimental unit.

Egg quality measurements were performed at the Laboratorio de Investigación en Nutrición y Alimentación de Aves (LINAA), Universidad Nacional Agraria La Molina; complete blood counts were conducted at the Veterinary Laboratory of Universidad Peruana Cayetano Heredia; and serum corticosterone analysis was performed at Arvetlab Laboratory (Lima, Peru).

To obtain the required data, eggs were collected daily, individually weighed, and counted to calculate the weekly laying rate for each treatment. External and internal egg quality parameters were evaluated during the first and last week of the trial. EW, AW, YW, and SW were recorded using an electronic balance (Henkel®, model ES-200211; capacity 300 g; precision 0.01 g). Linear measurements were performed using a digital caliper (Stainless Hardened; precision 0.01 mm) to measure EL, ED, AD, AH, YD, YH, and ST. Egg contents were carefully broken onto flat plastic platforms, providing a completely level surface to ensure accurate and repeatable measurements of albumen and yolk dimensions. Yolk color was evaluated using the Roche Yolk Color Fan (DSM®, 15-point scale). SW and ST were determined after washing the shells and air-drying them at room temperature for three days. Shell weight was measured using a portable digital pocket scale (Juwelier®, 200 g capacity, 0.01 g precision).

One day before the end of the trial, all birds were individually weighed and subjected to the tonic immobility test, which consisted of placing each quail on its back and recording the time taken to move or stand up. On the final day, seven birds per treatment were randomly selected for blood sampling. Before collection, quails were placed in a glass container containing cotton soaked with chloroform for anaesthesia. Subsequently, cardiac puncture was performed to withdraw 1 ml of blood using a tuberculin syringe for corticosterone and hematological analyses. Blood samples for corticosterone analysis were collected in serum (dry) tubes, whereas samples for complete blood counts were placed in EDTA tubes.

Haematocrit was determined using the microhematocrit method, and haemoglobin concentration was measured by the cyanmethemoglobin method. Total erythrocyte and leukocyte counts were performed using a Neubauer haemocytometer with Natt–Herrick solution. Differential leukocyte counts were obtained from blood smears stained with Wright–Giemsa, counting 100 leukocytes per slide under light microscopy, and the heterophil-to-lymphocyte (H:L) ratio was calculated from these differential counts. Serum corticosterone concentration was determined by enzyme-linked immunosorbent assay (ELISA) using standard laboratory procedures.

### Statistical analysis

Data normality was evaluated using the Kolmogorov–Smirnov test, and homogeneity of variances was assessed using Bartlett’s test. Data were analysed using a two-way analysis of variance (ANOVA) under a 2 × 3 factorial arrangement, considering VE level and stocking density as fixed factors. The main effects of VE and stocking density, as well as their interaction, were evaluated. When no significant interaction was detected, simple effects of each factor were analysed independently. Treatment means were compared using Tukey’s multiple comparison test, which controls the family-wise error rate and is appropriate for balanced designs when all pairwise comparisons among means are required. All statistical analyses were performed using Minitab® 22.

## Results

### Productive parameters

No interaction (*p* > 0.05) was observed between Valerian Extract (VE) and stocking density for body weight (BW), feed intake (FI), laying rate (LR), or egg mass (EM) during the seven weeks of evaluation (Table 2), indicating that the response of productive parameters to VE supplementation was consistent across stocking densities. Regarding main effects, stocking density significantly affected FI (*p* < 0.05), with birds housed individually consuming less feed (22.20 g) than those housed at two (23.32 g) or three birds per cage (23.66 g). The main effect of VE did not significantly affect BW, FI, or LR (*p* > 0.05); however, a tendency toward lower EM was observed in birds receiving VE (7.24 g/bird/day) compared with the control group (7.95 g/bird/day; *p* = 0.072).

**Table 2:** Productive performance parameters at week 7.

Item	BW (g)	FI (g)	LR (%)	EM (g/bird/d)
Main effect of Valerian extract				
0 mg/mL water	198.45	23.33	75.50	7.95
4 mg/ mL water	196.51	22.79	70.61	7.24
Main effect of density				
1 bird per cage	197.33	22.20^b^	71.80	7.46
2 birds per cage	195.10	23.32^a^	76.37	8.00
3 birds per cage	200.02	23.66^a^	70.99	7.34
Main effect of interaction Valerian extract x Density (treatments)				
T1	199.72	22.83	75.74	8.10
T2	197.86	23.36	73.93	7.75
T3	197.78	23.80	76.83	8.00
T4	194.93	21.58	67.86	6.82
T5	192.35	23.28	78.81	8.24
T6	202.25	23.51	65.16	6.67
SEM	1.44	0.19	1.79	0.21
P-values				
Valerian extract	0.505	0.154	0.153	0.072
Density	0.395	0.007	0.367	0.314
Valerian extract x Density	0.306	0.417	0.129	0.101

BW: body weight; FI: feed intake; LR: laying rate; EM = Egg Mass.

a,b Means within a column with different superscripts differ significantly (*p* < 0.05). SEM: Standard error of the mean.

### External egg characteristics

Shell thickness was significantly affected by both the interaction between factors (*p* < 0.05) and the main effect of VE (*p* < 0.05) (Table 3). Treatment T3 exhibited the greatest shell thickness (0.31 mm), which was higher than that observed in T1 (0.28 mm), T5 (0.27 mm), and T6 (0.28 mm), while being comparable to T2 (0.30 mm) and T4 (0.29 mm). As a main effect, birds receiving VE showed thinner shells (0.28 mm) compared with those in the control group (0.30 mm). No significant effects (*p* > 0.05) of VE, stocking density, or their interaction were detected for egg length, egg diameter, shell weight, or egg weight.

**Table 3:** External egg quality traits (egg dimensions, shell characteristics, and egg weight) of laying quails at week 7.

Item	EL (mm)	ED (mm)	SW (g)	ST (mm)	EW (g)
Main effect of Valerian extract					
0 mg/mL water	31.37	24.64	0.84	0.30^a^	10.51
4 mg/ mL water	31.31	24.55	0.83	0.28^b^	10.22
Main effect of density					
1 bird per cage	31.35	24.54	0.82	0.28	10.32
2 birds per cage	31.28	24.64	0.83	0.29	10.44
3 birds per cage	31.39	24.59	0.86	0.29	10.33
Main effect of interaction Valerian extract x Density (treatments)					
T1	31.67	24.76	0.83	0.28^bc^	10.68
T2	30.99	24.63	0.82	0.30^ab^	10.43
T3	31.44	24.51	0.86	0.31^a^	10.43
T4	31.02	24.31	0.82	0.29abc	9.97
T5	31.57	24.65	0.83	0.27^c^	10.46
T6	31.34	24.67	0.86	0.28^bc^	10.22
SEM	0.14	0.08	0.01	0.002	0.10
P-values					
Valerian extract	0.856	0.635	0.898	0.002	0.212
Density	0.949	0.916	0.362	0.130	0.876
Valerian extract x Density	0.307	0.466	0.968	0.046	0.511

EH: egg length; ED: egg diameter; SW: shell weight; ST: shell thickness; EW: egg weight.

a,b,c Means within a column with different superscripts differ significantly (*p* < 0.05). SEM: Standard error of the mean.

### Albumen and yolk characteristics

Albumen height was influenced by the interaction between VE and stocking density (*p* = 0.050) (Table 4), with T2 showing the highest albumen height (4.29 mm), whereas the remaining treatments ranged from 3.83 to 4.20 mm. No significant main effects of VE or stocking density were observed for albumen diameter, albumen weight, or Haugh units (*p* > 0.05).

**Table 4:** Albumen quality traits of laying quails at week 7.

Item	AD (mm)	AH (mm)	AW (g)	HU
Main effect of Valerian extract				
0 mg/mL water	36.92	4.13	5.82	88.54
4 mg/ mL water	37.82	4.02	5.67	87.83
Main effect of density				
1 bird per cage	37.62	4.16	5.80	88.74
2 birds per cage	36.93	4.06	5.69	87.99
3 birds per cage	37.57	4.01	5.74	87.81
Main effect of interaction Valerian extract x Density (treatments)				
T1	37.94	4.11^ab^	6.10	88.21
T2	35.29	4.29^a^	5.75	89.28
T3	37.52	3.98^ab^	5.61	88.13
T4	37.28	4.20^ab^	5.51	89.28
T5	38.56	3.83^b^	5.64	86.70
T6	37.63	4.03^ab^	5.88	87.50
SEM	0.35	0.05	0.07	0.32
P-values				
Valerian extract	0.228	0.320	0.387	0.309
Density	0.661	0.582	0.882	0.582
Valerian extract x Density	0.062	0.050	0.142	0.124

AD: albumen diameter; AH: albumen height; AW: albumen weight; HU: Haugh units.

a,b Means within a column with different superscripts differ significantly (*p* < 0.05). SEM: Standard error of the mean.

For yolk traits (Table 5), significant interactions were detected for yolk height (*p* < 0.05) and yolk colour (*p* < 0.05). Treatment T5 presented the greatest yolk height (9.88 mm), while T2 showed the lowest value (9.15 mm). Yolk colour was highest in T1 (4.00) and lowest in T4 (3.30). As main effects, VE significantly increased yolk diameter (23.86 vs. 22.78 mm; *p* < 0.05) and reduced yolk index (0.40 vs. 0.42; *p* < 0.05) compared with the control group. No significant effects (*p* > 0.05) were observed for yolk weight or yolk colour as main effects.

**Table 5:** Yolk quality traits of laying quails at week 7.

Item	YD (mm)	YH (mm)	YW (g)	YI	YC
Main effect of Valerian extract					
0 mg/mL water	22.78^b^	9.47	3.12	0.42^a^	3.78
4 mg/ mL water	23.86^a^	9.57	3.06	0.40^b^	3.60
Main effect of density					
1 bird per cage	23.07	9.58	3.10	0.42	3.65
2 birds per cage	23.42	9.52	3.12	0.41	3.68
3 birds per cage	23.46	9.46	3.06	0.41	3.73
Main effect of interaction Valerian extract x Density (treatments)					
T1	22.72	9.82^ab^	3.18	0.44	4.00^a^
T2	22.56	9.15^b^	3.05	0.42	3.45^b^
T3	23.04	9.43^ab^	3.13	0.41	3.90^a^
T4	23.41	9.34^ab^	3.01	0.40	3.30^b^
T5	24.29	9.88^a^	3.18	0.41	3.92^a^
T6	23.87	9.49^ab^	2.99	0.40	3.57^ab^
SEM	0.14	0.08	0.04	0.003	0.06
P-values					
Valerian extract	0.001	0.552	0.538	0.011	0.146
Density	0.581	0.875	0.852	0.424	0.860
Valerian extract x Density	0.273	0.031	0.311	0.284	0.001

YD: yolk diameter; YH: yolk height; YW: yolk weight; YI: yolk index; YC: yolk colour.

a,b Means within a column with different superscripts differ significantly (*p* < 0.05). SEM: Standard error of the mean.

### Stress indicators

A significant interaction between VE and stocking density was observed for the heterophil-to-lymphocyte (H:L) ratio (*p* < 0.05) (Table 6). Lower H:L ratios were recorded in T4 (1.08) and T5 (1.10) compared with T1 (2.32), indicating reduced physiological stress under these treatment combinations. As main effects, stocking density significantly influenced the H:L ratio (*p* < 0.05), with individually housed birds showing higher values (1.70) than those housed at two birds per cage (1.05), while birds housed at three per cage showed intermediate values (1.26). Valerian extract supplementation tended to reduce the H:L ratio (1.15 vs. 1.52; *p* = 0.078). Serum corticosterone concentration was significantly reduced by VE as a main effect (0.38 µg/dL) compared with the control group (0.62 µg/dL; *p* < 0.05). No significant effects (*p* > 0.05) were detected for tonic immobility time.

**Table 6:** Stress indicators of laying quails at week 7.

Item	CORT (µg/dL)	H:L ratio	TIT (s)
Main effect of Valerian extract			
0 mg/mL water	0.62^a^	1.52	17.16
4 mg/ mL water	0.38^b^	1.15	15.39
Main effect of density			
1 bird per cage	0.49	1.70^a^	14.30
2 birds per cage	0.48	1.05^b^	14.15
3 birds per cage	0.54	1.26^ab^	20.37
Main effect of interaction Valerian extract x Density (treatments)			
T1	0.58	2.32^a^	13.12
T2	0.53	1.00^b^	13.36
T3	0.75	1.24^ab^	25.00
T4	0.40	1.08^b^	15.48
T5	0.43	1.10^b^	14.94
T6	0.32	1.28^ab^	15.74
SEM	0.04	0.11	1.47
P-values			
Valerian extract	0.003	0.078	0.540
Density	0.778	0.036	0.148
Valerian extract x Density	0.173	0.015	0.199

CORT: corticosterone; H:L ratio: heterophil to lymphocyte ratio; TIT: tonic immobility time.

a,b Means within a column with different superscripts differ significantly (*p* < 0.05). SEM: Standard error of the mean.

### Haematological parameters

Significant interactions between VE and stocking density were detected for haematocrit (*p* < 0.05) and eosinophil percentage (*p* < 0.05) (Table 7). In contrast, monocyte percentage was significantly affected by stocking density as a main effect (*p* < 0.05), with birds housed individually showing higher monocyte levels (4.86 %) compared with those housed at three birds per cage (2.33 %), while birds housed at two per cage showed intermediate values (3.57 %). No significant main effects or interactions (*p* > 0.05) were observed for haemoglobin concentration, red blood cell count, white blood cell count, heterophils, or lymphocytes, indicating that VE supplementation and stocking density did not induce adverse alterations in overall haematological status.

**Table 7:** Haematological parameters of laying quails at week 7.

Item	Ht (%)	Hb (g/dL)	RBC (µl x 10 ^6^)	WBC (µl x 10 ^3^)	Heterophils (%)	Lymphocytes (%)	Monocytes (%)	Eosinophils (%)
Main effect of Valerian extract								
0 mg/mL water	41.48	7.95	3.14	12.96	54.00	37.52	3.75	4.76
4 mg/ mL water	42.42	7.78	3.38	11.25	46.84	45.23	3.43	4.67
Main effect of density								
1 bird per cage	44.29	7.64	3.44	10.68	53.57	37.00	4.86^a^	4.57
2 birds per cage	40.21	7.55	3.09	12.22	46.43	45.14	3.57^ab^	4.71
3 birds per cage	41.34	8.41	3.25	13.43	51.26	41.99	2.33^b^	4.86
Main effect of interaction Valerian extract x Density (treatments)								
T1	40.71^ab^	7.30	3.15	11.67	62.00	27.14	4.57	6.29^a^
T2	41.43^ab^	8.24	3.16	13.77	47.14	44.29	4.00	4.29abc
T3	42.29^ab^	8.30	3.11	13.45	52.86	41.14	2.67	3.71^bc^
T4	47.86^a^	7.98	3.73	9.68	45.14	46.86	5.14	2.86^c^
T5	39.00^b^	6.85	3.02	10.67	45.71	46.00	3.14	5.14abc
T6	40.40^ab^	8.52	3.38	13.41	49.67	42.83	2.00	6.00^ab^
SEM	0.85	0.22	0.10	0.68	2.08	2.16	0.36	0.39
P-values								
Valerian extract	0.547	0.712	0.261	0.223	0.082	0.065	0.664	0.897
Density	0.089	0.231	0.387	0.279	0.332	0.258	0.033	0.951
Valerian extract x Density	0.024	0.147	0.371	0.666	0.233	0.123	0.640	0.008

Ht: haematocrit; Hb: haemoglobin; RBC: red blood cells; WBC: white blood cells; Heterophils: heterophils; Lymphocytes: lymphocytes; Monocytes: monocytes; Eosinophils: eosinophils.

a,b,c Means within a column with different superscripts differ significantly (*p* < 0.05). SEM: Standard error of the mean.

## Discussion

In the present experiment, only feed intake (FI) was affected (*p* < 0.05) by stocking density; however, this response did not appear to be directly associated with crowding or physical competition for feed. Notably, birds housed individually consumed less feed despite unrestricted access to feeders and the absence of competitive pressure, suggesting that altered social context rather than space limitation per se contributed to changes in feeding behaviour. Quails are highly gregarious birds, and social facilitation is known to modulate several behavioural patterns, including feeding activity, spatial distribution, perching behaviour, and social synchronisation (Keeling and Duncan, 1988; Mills and Faure, 1989). Consequently, social isolation or reduced social interaction may act as a stressor in this species, potentially suppressing feeding motivation even under otherwise adequate management conditions (Wood-Gush, 1971). The productive performance results obtained in the present study are consistent with those reported by Marqués (2007) and Gravena et al. (2009). Marqués (2007) evaluated quails supplemented with increasing levels of chamomile, and Gravena et al. (2009) tested three levels of valerian added to quail diets; both studies found no significant effects on body weight (BW) or FI. Similarly, Esenbuga and Ekinci (2023) exposed laying hens to three cage densities (3, 4, and 5 hens/cage) and three herbal extracts (anise extract, black cumin seed extract, and thyme extract), observing no significant interactions between density and extract supplementation for BW, FI, or laying rate (LR). However, density and extracts analyzed independently did affect FI and LR (*p* < 0.05). In contrast, Dilawar et al. (2021) reported that *Mentha arvensis* and *Geranium thunbergii* extracts administered synergistically in various proportions in the diet of hens significantly increased FI, egg mass (EM), and LR compared to the control. Likewise, Hariono et al. (2025) found that Bandotan (*Ageratum conyzoides L*.) leaf extract added to drinking water improved FI, EM, and LR in quails relative to controls. Mustafa et al. (2024) demonstrated that a mixture of grapefruit (*Citrus paradisi*), olive (*Olea europaea*), and pomegranate (*Punica granatum*) extracts in drinking water at two doses across five ages enhanced EM and BW in quails compared with controls.

The absence of significant improvements in productive performance indicates that valerian extract (VE) mainly influenced stress-related physiological responses rather than directly enhancing production traits, which is consistent with its recognised pharmacological role as a mild sedative and anxiolytic agent. Notably, VE supplementation did not negatively affect BW, FI, LR, or EM across stocking densities, indicating that productive performance was maintained under the experimental conditions.

The lack of significant interactions between VE supplementation and stocking density for most productive and egg quality parameters suggests that the effects of VE may operate independently of the stocking density levels evaluated in the present study. A likely explanation is that the level of stress imposed by the tested stocking densities was not sufficiently intense to elicit a detectable interactive response. Under relatively moderate stress conditions, VE may modulate neuroendocrine stress pathways without producing measurable changes in performance traits.

In addition, the biological response to phytogenic additives is often dependent on stress intensity, dosage, and duration of supplementation. The use of a single inclusion level and a fixed administration period may have limited the detection of potential VE × stocking density interactions. Therefore, the absence of interaction effects should not be interpreted as a lack of biological activity of VE, but rather as an indication that its modulatory effects may become more evident under conditions of greater physiological challenge or alternative supplementation strategies.

Regarding egg characteristics, shell thickness (ST) was the only parameter affected by the interaction of factors and by the simple effect of VE. This partially agrees with Bandyopadhyay and Ahuja (1990), who reported that cage density does not affect egg length (EL) but may influence ST. The reduction in ST observed in birds receiving VE may be hypothetically associated with the reported calcium-antagonistic properties of VE. Experimental studies have demonstrated that hexanic and aqueous-methanolic extracts of *Valeriana spp*. exert calcium channel–blocking activity, primarily through inhibition of voltage-dependent l-type Ca²⁺ channels and suppression of intracellular Ca²⁺ release from the sarcoplasmic reticulum (Estrada et al., 2010; Bashir et al., 2011). l-type calcium channels play a key role in transcellular calcium transport and cellular calcium influx; therefore, their inhibition could theoretically reduce calcium availability for physiological processes involved in eggshell formation. In laying birds, adequate plasma calcium concentrations and efficient mobilisation of calcium are essential for eggshell mineralisation. Although intestinal absorption and hormonal regulation of calcium were not directly evaluated in the present study, the calcium channel–blocking activity reported for VE provides a plausible physiological mechanism by which VE supplementation may interfere with calcium mobilisation and deposition, resulting in thinner eggshells. Further studies are required to confirm whether these effects occur at the intestinal or medullary bone level in birds. These observations are partially supported by recent studies evaluating the effects of herbal extracts on egg characteristics. Esenbuga and Ekinci (2023) reported that in laying hens subjected to three cage densities (3, 4, and 5 hens/cage) and supplemented with anise extract, black cumin seed extract, and thyme extract, no significant differences were observed in egg weight (EW), shell weight (SW), or ST among treatments. Similarly, Hariono et al. (2025) found that administration of Bandotan (*Ageratum conyzoides L*.) leaf extract in quails did not affect EW, ST, or SW compared to controls. In contrast, Dilawar et al. (2021) observed that *Mentha arvensis* and *Geranium thunbergii* extracts increased EW relative to control birds, although ST differed by only approximately ±0.01 mm. Mustafa et al. (2024) reported significant differences in EW and ST when evaluating interactions between extract dose and bird age; however, analysis of individual extract effects showed no significant influence on these parameters. Collectively, these findings suggest that while some herbal extracts can modulate EW, SW and ST under specific conditions, the effects are generally small or context-dependent, supporting the notion that VE primarily influenced stress physiology rather than directly altering egg characteristics in the present study. Importantly, the magnitude of these changes is generally limited and may not translate into meaningful differences under commercial production conditions. From an applied perspective, the observed reduction in ST warrants careful consideration when VE is proposed as a stress-relieving supplement in laying systems. The calcium channel–blocking activity reported for *Valeriana spp*. may be dose-dependent and context-specific, indicating that supplementation strategies should balance stress mitigation benefits with mineral homeostasis. Therefore, future studies should further explore optimisation of inclusion level, duration of supplementation, and dietary calcium supply to minimize potential adverse effects on eggshell quality while preserving welfare-related benefits.

Regarding albumen characteristics, albumen height (AH) was influenced by the interaction between VE supplementation and stocking density, whereas the remaining albumen-related variables were not significantly affected. These findings are partially consistent with those reported by El-Tarabany et al. (2015), who observed that Haugh Units (HU) and AH were influenced by cage space, whereas albumen weight (AW) remained unaffected. Similarly, Esenbuga and Ekinci (2023) reported that HU were not affected by the interaction between herbal extracts and cage density, nor by stocking density alone; however, a modest reduction in HU was observed when herbal extracts were supplied independently, compared with the control group.

Regarding yolk traits, El-Tarabany et al. (2015) found significant effects of cage size on yolk height (YH), yolk diameter (YD), and yolk index (YI), but not on yolk weight (YW), which partially agrees with the present findings. In line with this, Esenbuga and Ekinci (2023) reported that YI and yolk colour (YC) were not influenced by the interaction between density and herbal extracts or by stocking density alone, although herbal extract supplementation slightly increased YC intensity and YI, with numerical values close to the control.

Other studies have shown variable responses of internal egg quality to herbal extract supplementation. Dilawar et al. (2021) reported statistically significant reductions in YC, YH, and HU in laying hens fed diets supplemented with different plant extracts compared with the control. In contrast, Hariono et al. (2025) observed no significant effects of Bandotan (*Ageratum conyzoides L*.) leaf extract supplied via drinking water on AW, YW, YC, or HU in quails. Mustafa et al. (2024) reported that the interaction between herbal extract supplementation and age significantly affected AH, YH, HU, and YC in laying quails; however, when the extract was analysed as a single factor, YD was not affected, and albumen diameter (AD) was influenced only by treatment interactions rather than by the extract itself.

Overall, these findings indicate that the effects of herbal extracts on albumen and yolk characteristics are inconsistent and highly context-dependent, varying according to extract type, dose, species, age, and environmental conditions. Moreover, changes in internal egg quality may also be indirectly associated with environmental stressors such as stocking density or thermal load, which can impair ovarian function either directly or through altered blood flow to the ovary (Baumgartner et al., 2008). Consequently, higher stocking densities and management-related stressors may exert a stronger influence on internal egg quality than herbal supplementation per se. Thus, the lack of consistent significant effects observed in the present study likely reflects biological variability rather than the absence of physiological responses. Under the conditions of the present study, birds were maintained under relatively controlled environmental conditions and moderate stocking densities, which may have limited the expression of extract-related effects on egg quality compared with studies conducted under higher physiological or environmental challenge.

The use of VE or stocking density did not show a clear relationship with tonic immobility time; however, VE supplementation showed a numerical tendency to reduce this variable. Our observations are in agreement with those of Gravena et al. (2009) and Ueoka (2020). Hazard et al. (2008) noted that further research is required to evaluate the effects of exogenous corticosterone administration on tonic immobility time, to better understand the relationship between corticosterone levels and immobility responses. These findings suggest that tonic immobility duration may not be directly dependent on corticosterone concentration in certain quail genotypes.

The H:L ratio was significantly affected by both the interaction of factors and the main effect of stocking density (*p* < 0.05). This aligns with Macari et al. (2002), who reported that under stress, birds exhibit increased release of corticotrophic hormones, leading to a reduction in lymphocytes and an increased heterophil-to-lymphocyte ratio. By contrast, corticosterone levels were significantly reduced by VE supplementation, supporting its stress-reducing potential. These findings are consistent with Esenbuga and Ekinci (2023), who reported that anise and black cumin extracts reduced corticosterone levels in laying hens under different cage densities. Similarly, Skomorucha and Sosnówka-Czajka (2013) observed lower corticosterone levels in broilers reared intensively when chamomile, lemon balm, or St John’s wort extracts were added to drinking water. Bry (1985) suggested that increases in plasma corticosterone during egg formation may not necessarily indicate direct metabolic involvement in egg production but could instead reflect an energetic stress response to reproduction.

The reduction in corticosterone levels and H:L ratio observed in quails receiving VE indicates a modulation of the physiological stress response rather than a direct enhancement of productive traits. Although this reduction was not accompanied by immediate improvements in egg production or egg quality, lower corticosterone concentrations and H:L ratios are widely recognised as indicators of improved welfare and reduced chronic stress load in poultry. Importantly, changes in stress biomarkers may precede measurable effects on productivity, particularly under conditions where baseline performance is already within normal physiological ranges. In this context, the stress-alleviating effects of VE may be attributed to the action of specific phytochemicals, as similarly reported in poultry supplemented with medicinal plant extracts under heat stress conditions (Mahasneh et al., 2024). Therefore, the observed modulation of stress biomarkers by VE supplementation should be interpreted primarily as a welfare-related benefit that may become more relevant under prolonged production cycles or under more challenging environmental or management conditions. Overall, the present results are consistent with a growing body of evidence indicating that several phytogenic additives, including chamomile, anise, black cumin, thyme, and valerian extracts, primarily exert stress-modulating effects in poultry, often reflected by reductions in corticosterone levels and H:L ratio, with limited or context-dependent effects on productive performance. From a practical perspective, these findings suggest that the application of VE may be more relevant in welfare-oriented production systems rather than as a strategy to directly enhance productive performance.

Aro et al. (2021) reported that higher stocking density increases stress in birds, leading to elevated eosinophil counts and erythrocyte sedimentation rate. These findings partially agree with our results, which show an increase in eosinophils and total white blood cell count as stocking density rises. Regarding haematocrit values, our results are consistent with those of Bourdon et al. (2021), who observed that quails exposed to moderate stress exhibited lower haematocrit concentrations without detrimental effects on performance. Our findings also indicate that VE does not affect haematological parameters, which is in agreement with Hidayatik et al. (2024), who found no significant effects on white blood cell counts, haematocrit, lymphocytes, or monocytes when garlic extract was supplemented in the drinking water of laying quails.

Despite the relevance of the present findings, some limitations should be acknowledged. Although environmental conditions were managed according to standard experimental practices, certain environmental and behavioural variables inherent to cage systems could not be fully controlled. In this context, physiological and productive responses to VE supplementation may be more pronounced under more challenging environmental or management conditions than those evaluated here. Therefore, the range of stocking densities applied in the present study may not have been sufficiently contrasting to induce strong stress responses, which could have limited the detection of potential interaction effects between VE and stocking density. More extreme conditions may be required to fully elucidate these interactions.

In addition, the physiological mechanisms underlying the effects of VE were not directly evaluated, as no direct measurements of calcium absorption, systemic calcium homeostasis, or bone metabolism were performed. Consequently, the proposed mechanisms linking VE to changes in eggshell thickness are based on indirect pharmacological evidence from previous experimental studies reporting calcium channel–blocking activity of *Valeriana spp*. extracts and should be interpreted as plausible explanations rather than direct causal inferences. Therefore, it cannot be excluded that alternative inclusion levels or longer supplementation periods might elicit detectable effects on productive performance or egg quality parameters.

Classical toxicological endpoints such as liver and kidney function markers, glucose, or lipid profiles were not assessed, as VE was administered at a dose considered safe and no clinical signs of toxicity or increased mortality were observed. Nevertheless, future studies could incorporate these parameters to further confirm the safety profile of prolonged VE supplementation. The experiment evaluated a single inclusion level of VE and a fixed supplementation period, which limits the ability to establish dose–response relationships and to assess potential long-term effects. As the biological response to phytogenic additives is often influenced by dosage, stress intensity, and duration of administration, the use of a single level may have constrained the detection of dose-dependent effects under the conditions of the present study. Therefore, future research should include multiple inclusion levels and extended supplementation periods to better characterize the dose–response relationship and to identify optimal strategies for VE use under different production and environmental conditions.

Serum corticosterone was measured at a single sampling time point, without accounting for potential circadian variations in glucocorticoid secretion, which may influence absolute hormone concentrations. Therefore, the observed differences should be interpreted as relative treatment effects rather than precise estimates of daily corticosterone profiles. Behavioural assessment was limited to tonic immobility time, which represents a validated but narrow indicator of fear response. More comprehensive behavioural observations, including locomotor activity, feeding behaviour patterns, social interactions, and abnormal behaviours, were beyond the scope of the present study.

Future studies should further explore different inclusion levels and administration periods of VE in quails, assess its effects under more challenging stress conditions such as thermal stress or increased social pressure, and evaluate whether the observed responses are consistent across different quail breeds or genetic lines, incorporating measurements related to calcium metabolism, stress pathways, and egg formation to better elucidate its mechanisms of action and practical applicability.

## Conclusion

Our results demonstrate that supplementation of valerian extract in drinking water reduces physiological stress indicators in laying quails without producing significant improvements in productivity or egg quality. These findings indicate that valerian extract primarily modulates stress-related physiological responses rather than directly enhancing productivity. Therefore, its practical application may be more relevant in the context of improving animal welfare under commercial production conditions. This study provides evidence that valerian extract supplementation may attenuate physiological stress indicators in laying quails under different stocking densities, warranting further investigation with expanded experimental designs and mechanistic approaches.

## CRediT authorship contribution statement

**Sidney Huapaya:** Writing – review & editing, Writing – original draft, Resources, Project administration, Methodology, Investigation, Conceptualization. **Daniel Huaringa:** Writing – review & editing, Writing – original draft, Software, Methodology, Investigation, Data curation. **Rubén Cespedes:** Resources, Methodology, Investigation. **Liliana Jiménez:** Validation, Methodology. **Otto Zea:** Validation, Supervision. **Iván Meza:** Methodology. **Carlos Vílchez-Perales:** Writing – review & editing, Validation, Supervision, Investigation, Conceptualization.

## Disclosures

The authors declare that they have no known competing financial interests or personal relationships that could have appeared to influence the work reported in this field study.
